# Supplementary material for: Drug repurposing for aging research using model organisms
Source: Aging Cell. 2017 Jun 16;16(5):1006–15. doi: 10.1111/acel.12626 (PMC5595691; doi:10.1111/acel.12626)
Supplement: Supplementary file 7 — Data S1 Zip‐Archive of all report cards. [file ACEL-16-1006-s007.zip › RC_MQY.pdf]

# MQY

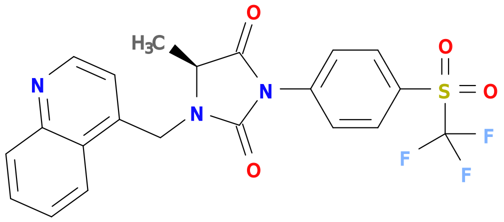


**Database identifiers**

ChEMBLCompound CHEMBL1738841

## Ranking

| | Rank | Score |
|---|---|---|
| Drosophila | 79/697 | 0.801 |
| C. elegans | 346/591 | 0.17 |

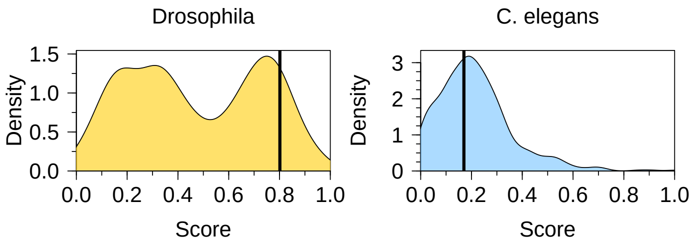


| | Ageing implication | Domain conservation | Binding site conservation | Binding affinity | Bioavailability | Lipinski | Promiscuity | Purchasability | Drug approval | **Total** |
|---|---|---|---|---|---|---|---|---|---|---|
| Drosophila | 1.0 | 0.931 | 1.0 | 0.957 | (0.9) | 0.0 | -0.0 | 0.0 | 0.0 | 0.801 |
| C. elegans | 1.0 | 0.87 | 0.98 | 0.957 | 0.209 | 0.0 | -0.0 | 0.0 | 0.0 | 0.17 |

## Names

No synonyms found

## Roles

ChEBI entry None has no roles

## Status

| | |
|---|---|
| Approved drug (according to ChEMBL) | No |
| Number of Rule of 5 violations | 0 |
| Binding affinity to original target in log units (RF-Score prediction) | 8.11 |
| Burns *C. elegans* bioavailability prediction | -6.01 |

## Compound Target Characteristics

### Insulin-like growth factor 1 receptor

Best gene implication in ageing for this target family came from gene P09208 annotated in UniProt release 2014_02. Annotation GO 8340 (determination of adult lifespan) was Inferred from Mutant

Phenotype

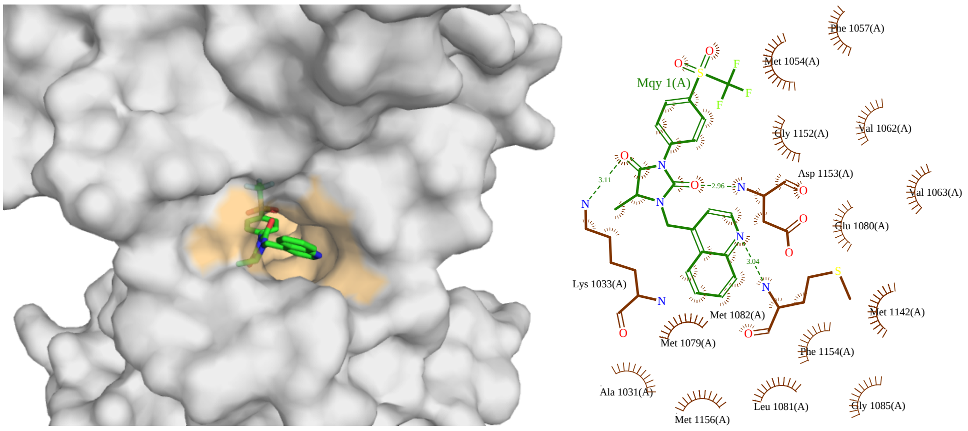

Mqy 1(A)
Phe 1057(A)
Met 1054(A)
Gly 1152(A)
Val 1062(A)
Asp 1153(A)
Val 1063(A)
Glu 1080(A)
Lys 1033(A)
Met 1082(A)
Met 1142(A)
Met 1079(A)
Phe 1154(A)
Ala 1031(A)
Met 1156(A)
Leu 1081(A)
Gly 1085(A)
3.11
2.96
3.04

| protein | amino acids contacts (binding site) | | | | | | | | | | | | | | | | | | | |
|---|---|---|---|---|---|---|---|---|---|---|---|---|---|---|---|---|---|---|---|---|
| PDB:3o23:chainA:P08069 | A | K | M | F | V | V | M | E | L | M | G | L | F | H | M | I | G | D | F | M |
| sp:P08069:IGF1R_HUMAN | A | K | M | F | V | V | M | E | L | M | G | L | F | H | M | I | G | D | F | M |
| tr:C9J5X1:C9J5X1_HUMAN | A | K | M | F | V | V | M | E | L | M | G | L | F | H | M | I | G | D | F | M |
| sp:P06213:INSR_HUMAN | A | K | M | F | V | V | M | E | L | M | G | L | F | H | M | I | G | D | F | M |
| sp:P24062:IGF1R_RAT | A | K | M | F | V | V | M | E | L | M | G | L | F | H | M | I | G | D | F | M |
| tr:Q3UVJ3:Q3UVJ3_MOUSE | A | K | M | F | V | V | M | E | L | M | G | L | F | H | M | I | G | D | F | M |
| tr:E9QNX9:E9QNX9_MOUSE | A | K | M | F | V | V | M | E | L | M | G | L | F | H | M | I | G | D | F | M |
| sp:P15208:INSR_MOUSE | A | K | M | F | V | V | M | E | L | M | G | L | F | H | M | I | G | D | F | M |
| sp:Q60751:IGF1R_MOUSE | A | K | M | F | V | V | M | E | L | M | G | L | F | H | M | I | G | D | F | M |
| tr:M4TKR7:M4TKR7_MOUSE | A | K | M | F | V | V | M | E | L | M | G | L | F | H | M | I | G | D | F | M |
| tr:G7H807:G7H807_DROME | A | K | M | F | V | V | M | E | L | M | G | L | F | H | M | I | G | D | F | M |
| tr:D0UY96:D0UY96_DROME | A | K | M | F | V | V | M | E | L | M | G | L | F | H | M | I | G | D | F | M |
| tr:D0UY86:D0UY86_DROME | A | K | M | F | V | V | M | E | L | M | G | L | F | H | M | I | G | D | F | M |
| tr:D0UY84:D0UY84_DROME | A | K | M | F | V | V | M | E | L | M | G | L | F | H | M | I | G | D | F | M |
| tr:D0UY85:D0UY85_DROME | A | K | M | F | V | V | M | E | L | M | G | L | F | H | M | I | G | D | F | M |
| tr:D0UY80:D0UY80_DROME | A | K | M | F | V | V | M | E | L | M | G | L | F | H | M | I | G | D | F | M |
| tr:D0UY81:D0UY81_DROME | A | K | M | F | V | V | M | E | L | M | G | L | F | H | M | I | G | D | F | M |
| tr:D0UY88:D0UY88_DROME | A | K | M | F | V | V | M | E | L | M | G | L | F | H | M | I | G | D | F | M |
| tr:D0UY89:D0UY89_DROME | A | K | M | F | V | V | M | E | L | M | G | L | F | H | M | I | G | D | F | M |
| tr:D0UYA1:D0UYA1_DROME | A | K | M | F | V | V | M | E | L | M | G | L | F | H | M | I | G | D | F | M |
| tr:D0UYA0:D0UYA0_DROME | A | K | M | F | V | V | M | E | L | M | G | L | F | H | M | I | G | D | F | M |
| tr:D0UYA6:D0UYA6_DROME | A | K | M | F | V | V | M | E | L | M | G | L | F | H | M | I | G | D | F | M |
| tr:D0UY91:D0UY91_DROME | A | K | M | F | V | V | M | E | L | M | G | L | F | H | M | I | G | D | F | M |
| tr:D0UY97:D0UY97_DROME | A | K | M | F | V | V | M | E | L | M | G | L | F | H | M | I | G | D | F | M |
| tr:D0UY78:D0UY78_DROME | A | K | M | F | V | V | M | E | L | M | G | L | F | H | M | I | G | D | F | M |
| tr:D0UY95:D0UY95_DROME | A | K | M | F | V | V | M | E | L | M | G | L | F | H | M | I | G | D | F | M |
| tr:D0UY94:D0UY94_DROME | A | K | M | F | V | V | M | E | L | M | G | L | F | H | M | I | G | D | F | M |
| tr:D0UY75:D0UY75_DROME | A | K | M | F | V | V | M | E | L | M | G | L | F | H | M | I | G | D | F | M |
| tr:D0UY77:D0UY77_DROME | A | K | M | F | V | V | M | E | L | M | G | L | F | H | M | I | G | D | F | M |
| tr:D0UY70:D0UY70_DROME | A | K | M | F | V | V | M | E | L | M | G | L | F | H | M | I | G | D | F | M |
| tr:D0UYA5:D0UYA5_DROME | A | K | M | F | V | V | M | E | L | M | G | L | F | H | M | I | G | D | F | M |
| tr:D0UYA9:D0UYA9_DROME | A | K | M | F | V | V | M | E | L | M | G | L | F | H | M | I | G | D | F | M |
| tr:D0UY99:D0UY99_DROME | A | K | M | F | V | V | M | E | L | M | G | L | F | H | M | I | G | D | F | M |
| tr:D0UYA4:D0UYA4_DROME | A | K | M | F | V | V | M | E | L | M | G | L | F | H | M | I | G | D | F | M |
| tr:D0UYA8:D0UYA8_DROME | A | K | M | F | V | V | M | E | L | M | G | L | F | H | M | I | G | D | F | M |
| tr:D0UY98:D0UY98_DROME | A | K | M | F | V | V | M | E | L | M | G | L | F | H | M | I | G | D | F | M |
| tr:D0UYA7:D0UYA7_DROME | A | K | M | F | V | V | M | E | L | M | G | L | F | H | M | I | G | D | F | M |
| tr:C7C412:C7C412_DROME | A | K | M | F | V | V | M | E | L | M | G | L | F | H | M | I | G | D | F | M |
| tr:C7C413:C7C413_DROME | A | K | M | F | V | V | M | E | L | M | G | L | F | H | M | I | G | D | F | M |
| tr:C7C410:C7C410_DROME | A | K | M | F | V | V | M | E | L | M | G | L | F | H | M | I | G | D | F | M |
| tr:C7C411:C7C411_DROME | A | K | M | F | V | V | M | E | L | M | G | L | F | H | M | I | G | D | F | M |
| tr:C7C414:C7C414_DROME | A | K | M | F | V | V | M | E | L | M | G | L | F | H | M | I | G | D | F | M |
| tr:C7C415:C7C415_DROME | A | K | M | F | V | V | M | E | L | M | G | L | F | H | M | I | G | D | F | M |
| tr:C7C417:C7C417_DROME | A | K | M | F | V | V | M | E | L | M | G | L | F | H | M | I | G | D | F | M |
| sp:P09208:INSR_DROME | A | K | M | F | V | V | M | E | L | M | G | L | F | H | M | I | G | D | F | M |
| tr:C7C416:C7C416_DROME | A | K | M | F | V | V | M | E | L | M | G | L | F | H | M | I | G | D | F | M |
| sp:Q968Y9:INSR_CAEEL | A | K | M | F | I | V | M | E | M | M | G | L | F | H | M | I | G | D | F | M |

| protein | whole protein ident | simil | domain-based ident | simil | contact-based ident | simil |
|---|---|---|---|---|---|---|
| PDB:3o23:chainA:P08069 | 1.0 | 1.0 | 1.0 | 1.0 | 1.0 | 1.0 |
| sp:P08069:IGF1R_HUMAN | 1.0 | 1.0 | 1.0 | 1.0 | 1.0 | 1.0 |
| tr:C9J5X1:C9J5X1_HUMAN | 1.0 | 1.0 | 1.0 | 1.0 | 1.0 | 1.0 |
| sp:P06213:INSR_HUMAN | 0.56 | 0.83 | 0.83 | 0.95 | 1.0 | 1.0 |
| sp:P24062:IGF1R_RAT | 0.96 | 0.99 | 0.98 | 0.99 | 1.0 | 1.0 |
| tr:Q3UVJ3:Q3UVJ3_MOUSE | 0.65 | 0.67 | 0.97 | 0.99 | 1.0 | 1.0 |
| tr:E9QNX9:E9QNX9_MOUSE | 0.96 | 0.99 | 0.97 | 0.99 | 1.0 | 1.0 |
| sp:P15208:INSR_MOUSE | 0.56 | 0.84 | 0.82 | 0.95 | 1.0 | 1.0 |
| sp:Q60751:IGF1R_MOUSE | 0.96 | 0.99 | 0.97 | 0.99 | 1.0 | 1.0 |
| tr:M4TKR7:M4TKR7_MOUSE | 0.56 | 0.83 | 0.82 | 0.95 | 1.0 | 1.0 |
| tr:G7H807:G7H807_DROME | 0.13 | 0.22 | 0.65 | 0.86 | 1.0 | 1.0 |
| tr:D0UY96:D0UY96_DROME | 0.23 | 0.48 | 0.65 | 0.86 | 1.0 | 1.0 |
| tr:D0UY86:D0UY86_DROME | 0.23 | 0.48 | 0.65 | 0.86 | 1.0 | 1.0 |
| tr:D0UY84:D0UY84_DROME | 0.23 | 0.48 | 0.65 | 0.86 | 1.0 | 1.0 |
| tr:D0UY85:D0UY85_DROME | 0.23 | 0.48 | 0.65 | 0.86 | 1.0 | 1.0 |
| tr:D0UY80:D0UY80_DROME | 0.23 | 0.48 | 0.65 | 0.86 | 1.0 | 1.0 |
| tr:D0UY81:D0UY81_DROME | 0.23 | 0.48 | 0.65 | 0.86 | 1.0 | 1.0 |
| tr:D0UY88:D0UY88_DROME | 0.23 | 0.48 | 0.65 | 0.86 | 1.0 | 1.0 |
| tr:D0UY89:D0UY89_DROME | 0.23 | 0.48 | 0.65 | 0.86 | 1.0 | 1.0 |
| tr:D0UYA1:D0UYA1_DROME | 0.23 | 0.48 | 0.65 | 0.86 | 1.0 | 1.0 |
| tr:D0UYA0:D0UYA0_DROME | 0.23 | 0.48 | 0.65 | 0.86 | 1.0 | 1.0 |
| tr:D0UYA6:D0UYA6_DROME | 0.23 | 0.48 | 0.65 | 0.86 | 1.0 | 1.0 |
| tr:D0UY91:D0UY91_DROME | 0.23 | 0.48 | 0.65 | 0.86 | 1.0 | 1.0 |
| tr:D0UY97:D0UY97_DROME | 0.22 | 0.48 | 0.65 | 0.86 | 1.0 | 1.0 |
| tr:D0UY78:D0UY78_DROME | 0.23 | 0.48 | 0.65 | 0.86 | 1.0 | 1.0 |
| tr:D0UY95:D0UY95_DROME | 0.23 | 0.48 | 0.65 | 0.86 | 1.0 | 1.0 |
| tr:D0UY94:D0UY94_DROME | 0.23 | 0.48 | 0.65 | 0.86 | 1.0 | 1.0 |
| tr:D0UY75:D0UY75_DROME | 0.23 | 0.48 | 0.65 | 0.86 | 1.0 | 1.0 |
| tr:D0UY77:D0UY77_DROME | 0.23 | 0.48 | 0.65 | 0.86 | 1.0 | 1.0 |
| tr:D0UY70:D0UY70_DROME | 0.23 | 0.48 | 0.65 | 0.86 | 1.0 | 1.0 |
| tr:D0UYA5:D0UYA5_DROME | 0.23 | 0.48 | 0.65 | 0.86 | 1.0 | 1.0 |
| tr:D0UYA9:D0UYA9_DROME | 0.23 | 0.48 | 0.65 | 0.86 | 1.0 | 1.0 |
| tr:D0UY99:D0UY99_DROME | 0.23 | 0.48 | 0.65 | 0.86 | 1.0 | 1.0 |
| tr:D0UYA4:D0UYA4_DROME | 0.23 | 0.48 | 0.65 | 0.86 | 1.0 | 1.0 |
| tr:D0UYA8:D0UYA8_DROME | 0.23 | 0.48 | 0.65 | 0.86 | 1.0 | 1.0 |
| tr:D0UY98:D0UY98_DROME | 0.23 | 0.48 | 0.65 | 0.86 | 1.0 | 1.0 |
| tr:D0UYA7:D0UYA7_DROME | 0.22 | 0.48 | 0.65 | 0.86 | 1.0 | 1.0 |
| tr:C7C412:C7C412_DROME | 0.22 | 0.46 | 0.65 | 0.86 | 1.0 | 1.0 |
| tr:C7C413:C7C413_DROME | 0.22 | 0.46 | 0.65 | 0.86 | 1.0 | 1.0 |
| tr:C7C410:C7C410_DROME | 0.22 | 0.46 | 0.65 | 0.86 | 1.0 | 1.0 |
| tr:C7C411:C7C411_DROME | 0.22 | 0.46 | 0.65 | 0.86 | 1.0 | 1.0 |
| tr:C7C414:C7C414_DROME | 0.22 | 0.46 | 0.65 | 0.86 | 1.0 | 1.0 |
| tr:C7C415:C7C415_DROME | 0.22 | 0.46 | 0.65 | 0.86 | 1.0 | 1.0 |
| tr:C7C417:C7C417_DROME | 0.21 | 0.46 | 0.65 | 0.86 | 1.0 | 1.0 |
| sp:P09208:INSR_DROME | 0.22 | 0.46 | 0.65 | 0.86 | 1.0 | 1.0 |
| tr:C7C416:C7C416_DROME | 0.21 | 0.46 | 0.65 | 0.86 | 1.0 | 1.0 |
| sp:Q968Y9:INSR_CAEEL | 0.2 | 0.52 | 0.46 | 0.79 | 0.9 | 0.98 |

### InR (FBgn0013984) associated phenotypes

RU486 conditional, aging defective, body size defective, cell autonomous, cell number defective, cell size defective, chemical sensitive, circadian rhythm defective, conditional, decreased cell number, decreased cell size, developmental rate defective, dominant, feeding behavior defective, heat sensitive, hyperplasia, increased cell size, lethal - all die before end of second instar larval stage, long lived, mating defective, melanotic mass phenotype, neuroanatomy defective, neurophysiology defective, oxidative stress response defective, partially, partially lethal, partially lethal - majority die, short lived, size defective, sleep defective, small body, somatic clone, some die during pupal stage, some die during second instar larval stage, starvation stress response defective, stress response defective

(Information from FlyBase)

### InR (UniProt:P09208) annotation

**Function**: Has a ligand-stimulated tyrosine-protein kinase activity. Required for cell survival. Reg-

ulates body size and organ size by altering cell number and cell size in a cell- autonomous manner. Involved in the development of the embryonic nervous system, and is necessary for axon guidance and targeting in the visual system. Also plays a role in life-span determination. (PubMed:10455177, PubMed:11250149, PubMed:11292875, PubMed:12702880, PubMed:7628438, PubMed:8603594).
**Cofactor**: Mn(2+)
**Enzyme regulation**: Autophosphorylation activates the kinase activity.
**Subunit**: Tetramer of 2 alpha and 2 beta chains linked by disulfide bonds. The alpha chains contribute to the formation of the ligand- binding domain, while the beta chains carry the kinase domain. When autophosphorylated, the beta-subunit binds the SH2 and SH3 domains of the adapter protein Dock. The beta subunit also binds and tyrosine phosphorylates the insulin receptor substrate Chico.
**Subcellular location**: Membrane; Single-pass type I membrane protein.
**Tissue specificity**: Widely distributed throughout the embryo. Expressed at high levels in the embryonic nervous system. Larval expression is limited to the nervous system and all imaginal disks. Expressed at high levels in the adult nervous system and ovaries. (PubMed:2454394, PubMed:7628438).
**Developmental stage**: Expressed throughout development. Embryonic expression is most prominent 8 to 12 hours after egg laying. (PubMed:2454394, PubMed:3014506).
**Ptm**: The 280 kDa proreceptor is proteolytically processed to form a 120 kDa alpha subunit and a 170 kDa beta subunit. The beta subunit undergoes cell-specific cleavage to generate a 90 kDa beta subunit and a free 60 kDa C-terminal subunit. Both the 90 kDa and the 170 kDa beta subunits can assemble with the alpha subunits to form mature receptors. (PubMed:7628438).
**Ptm**: Autophosphorylated on tyrosine residues in response to exogenous insulin. (PubMed:18327897).
**Ptm**: Phosphorylation of Tyr-1354 is required for Chico-binding.

(Information from UniProt)

**daf-2 (WBGene00000898) associated phenotypes**
DMPP resistant, L2 larval development variant, aging variant, anoxia resistant, autophagy variant, brood size variant, carbohydrate metabolism variant, carbon dioxide avoidance variant, dauer arrest variant, dauer constitutive, dauer formation variant, diet induced life span variant, drug response variant, egg laying variant, extended life span, fat content increased, gene expression level high, gene expression variant, hormetic temperature induced life span extension defective, larval lethal, lethal, lipid composition variant, lipid metabolism variant, lipid synthesis increased, mRNA expression variant, mRNA levels increased, mate searching variant, mianserin resistant, no dauer recovery, organ senescence variant, organism UV resistant, organism dessication response variant, organism hypersensitive dessication, organism oxidative stress response variant, organism physiology variant, organism starvation response variant, pathogen resistance increased, protein expression increased, protein phosphorylation increased, protein phosphorylation reduced, quiescence variant, reduced brood size, reproductive longevity extended, response to food variant, roaming reduced, spontaneous spicule protraction, sterile, temperature influenced life span variant, thermotolerance increased, transgene expression increased, transgene expression reduced, transgene subcellular localization variant, yolk synthesis variant

(Information from WormBase)

**daf-2 (UniProt:Q968Y9) annotation**
**Function**: An insulin receptor-like protein which regulates metabolism, controls longevity and prevents developmental arrest at the dauer stage. Binding of INS family members may either stimulate, or antagonize, association of the receptor with downstream mediators such as pdk-1 and age-1. Required for the response to environmental stimuli such as food, pheromone, and temperature. Role in immune function and pathogen resistance. (PubMed:11274053, PubMed:18782349, PubMed:9252323, PubMed:9790527).
**Cofactor**: Mn(2+) Evidence=(UniProtKB:P06213);

**Enzyme regulation**: Autophosphorylation activates the kinase activity (By similarity). Interaction with shc-1 may inhibits its activity (PubMed:18832074). (UniProtKB:P06213, PubMed:18832074).
**Subunit**: Tetramer of 2 alpha and 2 beta chains linked by disulfide bonds. The alpha chains contribute to the formation of the ligand- binding domain, while the beta chains carry the kinase domain (By similarity). Interacts (via cytoplasmic domain) with shc-1 (PID domain) (PubMed:18832074). (UniProtKB:P06213, PubMed:18832074).
**Subcellular location**: Membrane (UniProtKB:P06213); Single-pass type I membrane protein (UniProtKB:P06213).
**Disruption phenotype**: Accumulation of fat, pigmented intestine, increased life span, increased dauer formation and increased resistance to pathogens. Severe loss of function mutants display recessive early embryonic lethality. (PubMed:11274053, PubMed:18245374, PubMed:18782349, PubMed:9252323, PubMed:9790527).

(Information from UniProt)
